# Sensitivity and Accuracy of Dielectric Measurements of Liquids Significantly Improved by Coupled Capacitive-Dependent Quartz Crystals

**DOI:** 10.3390/s21103565

**Published:** 2021-05-20

**Authors:** Vojko Matko, Miro Milanovič

**Affiliations:** Faculty of Electrical Engineering and Computer Science, University of Maribor, Koroška c. 46, 2000 Maribor, Slovenia; miro.milanovic@um.si

**Keywords:** permittivity, liquid, capacitive-dependent quartz crystal, temperature compensation

## Abstract

A method to measure complex permittivity of liquids by using a capacitive-dependent quartz crystal and two quartz oscillators for temperature compensation in the frequency range of 4–10 MHz is described. Complex permittivity can be detected with high precision and sensitivity through a small change of capacitance and conductance, because a change in reactance in series with the quartz crystal impacts its resonant oscillation frequency. The temperature compensation in the range below 0.1 ppm is achieved by using two quartz oscillators that are made of elements of the same quality and have a temperature–frequency pair of quartz crystals. With the help of a reference oscillator, measurements of frequency are more accurate, because the frequency difference is in the kHz region, which also enables further processing of the signal by a microcontroller. With a proper calibration, the accuracy of this highly sensitive quartz crystal method is ±0.05%, which is an order of magnitude lower than that for a capacitance method without quartz crystals. The improved accuracy is of significant importance in the field of power engineering to monitor coolants and lubricants, oils, liquid fuels and other liquids, the dielectric properties of which are crucial for proper operation of devices.

## 1. Introduction

Characterization of the dielectric properties of materials, such as relative permittivity, conductivity and loss tangent, are of a great importance for a variety of applications. Accurate measurements of these characteristics can provide scientists with valuable information on the suitability of individual materials for the intended use and an enhanced product quality control. Next to the molecular structure of the material, the complex relative permittivity also depends on frequency, temperature, humidity and pressure. This is why numerous methods for measurements of the dielectric properties in various liquids, powders and solid materials in different frequency ranges [1,2,3,4,5] have been developed. Measurements of conductivity of liquids present an important tool for the analysis of binary liquids/electrolyte mixtures and to determine critical points of various liquid mixtures. It is also widely used for determination of the pollution of oils, fuels and lubricants in power engineering [6,7] and for measurements of dielectric properties in bio-applications [1,5,8]. Measurements of the dielectric characteristics are of particular importance in monitoring and controlling of liquids in situations where quality is a critical factor [9,10].

There are many different permittivity measurement methods, each having its advantages and drawbacks. These methods are classified into a few common types, including resonant cavity [11,12,13], coaxial probe [8,14,15], transmission line [16,17,18] and parallel plate capacitor [19,20,21,22]. In [23,24,25], a microwave resonator is calibrated with materials of known dielectric properties, usually with organic solvents such as methanol, ethanol, etc. The methods described in [23,24,25] provide a high loss tangent resolution over a measurement frequency range from 50 MHz to over 100 GHz. By adding a waveguide, permittivity can be measured even without a resonator, simply by placing the material (solid or liquid) in the waveguide, which thus gains an additional function of the sample holder. The dielectric constant and the loss tangent are calculated from the measurement of the reflection and transmission of the waveguide. Such a method is simpler, but compared to the method with a resonator, its accuracy is lower. The method with an open coaxial probe [10,26,27] is used for dielectric measurements of agricultural products and can be used for testing of liquid, soft and solid materials. The drawback of the method is in its lower accuracy below 200 MHz and, in general, for materials which have low dielectric constants and loss factors. We also point out a parallel plate method [28,29,30]. This is a standardized three-terminal method (ASTM-D150 [30,31]) in which a thin sample of the studied material is placed between capacitor plates. An impedance analyzer is used to measure the series capacitance and resistance, from which the dielectric constant and the loss tangent of the material can be calculated. Depending on the experimental setup, the method can achieve high accuracy at frequencies below 100 MHz.

This article proposes a new enhanced method for the measurement of permittivity of liquid samples by using a capacitive-dependent quartz crystal and an insulated capacitor which is fixed on a glass test tube. The novelty introduced by this method is in the impact of the capacitance changes, which are due to changes in the liquid permittivity, on the quartz crystal’s resonant oscillation frequency. This allows highly sensitive and precise measurements of the complex permittivity. We present the uncertainty of the proposed measurement method as well as calibration of the measuring system with the known dielectric properties of liquids. The advantage of the proposed measurement method is in its high sensitivity and its low cost compared to the above-mentioned methods, especially the ones that use high-cost impedance analyzers for determination of the relative permittivity and conductivity of liquids.

## 2. Materials and Methods

To measure dielectric properties, we use a capacitive method. Capacitance techniques for measurements of permittivity are useful in the frequency range from 1 kHz to 10 MHz. The drawbacks of the capacitance techniques are fringing fields near the edges of the capacitor plates and, at low frequencies, electrode polarization. The capacitance for a parallel plate capacitor with no fringing fields is given by

(1) $$C=\frac{\varepsilon_{0}\varepsilon_{r}}{d}A,$$

where $\varepsilon_{0}$ is the permittivity of free space, A is the capacitor plate area, d is the distance between the plates, and relative permittivity, $\varepsilon_{r}$, affects the amount of electric energy stored in the material. The conductance (G) of the capacitor at a low frequency (f<10 MHz) is given by [1,2,3]:

(2) $$G=\omega \frac{\varepsilon_{0}\varepsilon^{\prime \prime }}{d}A\;,$$

where ω is the angular frequency, and the dielectric loss, $\varepsilon^{\prime \prime }$, determines the loss factor, which is a measure of dissipation of electromagnetic energy in the material. The loss tangent (tanδ) is expressed as:

(3) $$\tan\delta =\frac{\varepsilon^{\prime \prime }}{\varepsilon_{r}}=\frac{G}{\omega C}\;.$$

The complex permittivity ($\varepsilon^{*}$) consists of the real and imaginary part [1,3,10]:

(4) $$\varepsilon^{*}=\varepsilon_{r}-j\varepsilon^{\prime \prime }=\frac{C-\frac{jG}{\omega }}{C_{air}-\frac{jG_{air}}{\omega }}\;,$$

where $C_{air}$ and $G_{air}$ are the capacitance and conductance of an empty glass test tube, respectively.

Table 1 provides a comparison of the advantages and measurement uncertainties of other measurement methods in relation to the existing capacitance method for the permittivity measurement. For a capacitance method, a typical measurement uncertainty is ±1% when measuring the real part of permittivity and $\pm 5\times 10^{-5}$ when measuring tanδ. In what follows, we show that by the improved method presented in this article, the measurement uncertainty is reduced to ±0.05% for the relative permittivity, with no significant change in the uncertainty of the loss tangent.

## 3. Results

### 3.1. Experimental Setup

**Figure 1:**
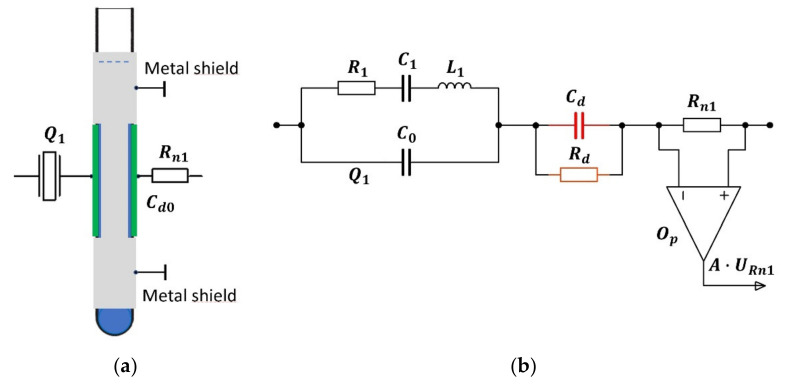

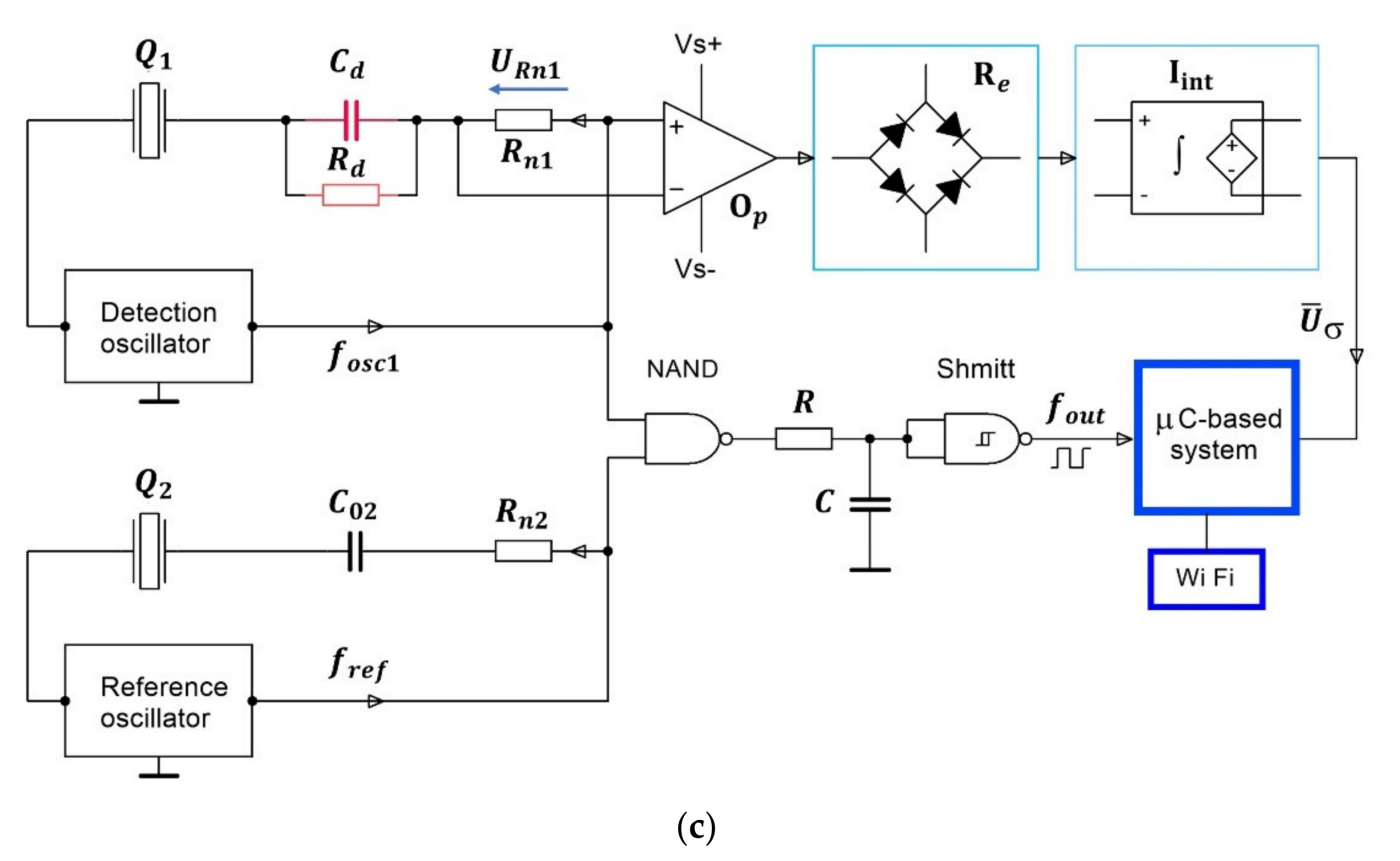
(**a**) The measuring part of the experimental setup: $C_{d0}$ is a capacitance of an empty glass tube, $Q_{1}$ is the quartz and $R_{n1}$ is a resistance of the series resistor. (**b**) A substitute electrical circuit from (**a**) with an operational amplifier added for the conductivity measurements. (**c**) Dielectric measurement principle by capacitive-dependent quartz crystal and two oscillators with similar crystal temperature–frequency characteristics.

The experimental setup (see Figure 1) for the measurement of complex permittivity consists of a glass test tube with capacitance, $C_{01}$, and quartz crystal oscillator. The capacitor (metal plates with dimensions 5×0.5 cm) is fixed on the external part of the glass test tube (Figure 1a) with a height of 12 cm, diameter 1.2 cm and the glass thickness of 1 mm [32]. The rest of the glass test tube is covered by a metal layer which acts as a shield to reduce the fringing field effect [1,2,3,30]. Equations (1)–(4) relate to this part of the experimental setup. Even though the actual capacitor is not a parallel plate capacitor, the approximation can be used to predict the trends, while the calibration of the sensor (given in Section 3.4) takes care of all the effects that are not described by this simplified theoretical consideration.

The substitute scheme for the experimental setup consisting of a glass test tube with a capacitor connected in series with a quartz crystal and resistance, $R_{n1}$, is shown in Figure 1b, where the left part of the scheme shows an equivalent electrical circuit of the quartz crystal. The capacitor with capacitance $C_{d0}$ represents the empty glass test tube, while $C_{d}$ and $R_{d}$ are the capacitance and resistance of the test tube filled with the measured liquid, respectively. The resistor with resistance $R_{n1}$ and operational amplifier $O_{p}$ are intended for the measurement of the conductance of the liquid under test at the frequency at which the quartz oscillator oscillates. 

The material from which the glass test tube is made plays an important role. It is essential that it has as high relative permittivity as possible. Table 2 displays values of relative permittivity and loss tangent for various types of glass at different frequencies. The best selection proved to be the use of an iron-sealing glass because it has the highest value of relative permittivity and the lowest value of the loss tangent up 100 MHz.

When measurements are performed on conducting liquids, one has to take into account ionic conductivity and electrode polarization, because the conducting ions collect on the electrodes, where they form a double layer with a very high capacitance. This double layer can be considered as a capacitor connected in series with the tested sample. For this reason, the measured relative permittivity is greater than the relative permittivity of the tested sample. A way of minimizing the effects of the electrode polarization is to coat the capacitor plates with a platinum black [34] or by using a four-probe capacitor system [35]. The four-probe capacitance technique measures the voltage drop away from the plates and thus avoids the double layer. In the method proposed here, we reduce the electrode polarization by placing the capacitor plates on the external surface of the glass test tube, as shown in Figure 1a. In addition, the experimentally used frequencies are 4 and 10 MHz, while the electrode polarization effect is most important at lower frequencies (few kHz to 100 kHz). The selection of the iron-sealing glass test tube with very low value of tangent loss additionally reduces the electrode polarization.

### 3.2. Crystal Temperature–Frequency Characteristics Compensation

The relative permittivity of the liquid is measured by the detection oscillator, while the reference oscillator is used for the temperature–frequency compensation of the temperature influence on the detection oscillator (Figure 1c). The temperature compensation is achieved by two quartz oscillators that are made in the same way (with elements of the same quality) and have a temperature–frequency pair of quartz crystals (produced by the crystal producer—Statek). The right part of the circuit in Figure 1c is used to convert the frequency, $f_{osc1}$, of the detection oscillator, which is in the MHz range, into a kHz frequency range. In this way, the precision of the measurement is increased by several orders of magnitude. In the case of an AT-cut crystal with a frequency change of ±1 ppm in the temperature range T=0–40 °C, the two oscillators have approximately the same frequency (≅ 4 MHz) [32,36,37]. The quartz crystal data are $L_{1}=158.314\;\mathrm{mH}$, $C_{1}=10\;\mathrm{fF}$, $R_{1}=10\;\Omega$, $C_{0}=2\;\mathrm{pF}$, and quality Q=153 k (measured by a HP4194A impedance analyzer (Hewlett Packard/Agilent)). The frequency difference, $f_{out}$, between both oscillators is set to ≅2 kHz when the glass test tube is empty. To achieve as equal impedance oscillation conditions as possible for both oscillators, we chose the capacitance, $C_{02}$, of the capacitor in the reference oscillator to be approximately equal to the capacitance of an empty glass tube, the value of which is estimated by assuming a parallel plate capacitor partially filled by glass and partially by air (see Section 3.4): $C_{d0}≅\;C_{02}=0.23\;\mathrm{pF}$. The resistance $R_{n2}$ of the resistor in the reference circuit is matched to the resistance $R_{n1}$ in the detection oscillator: $R_{n1}=R_{n2}=1.000\;\Omega$. The capacitance of the test tube filled by liquid, $C_{d}$, and resistance, $R_{d}$, depend on a liquid under a test and cannot be temperature-compensated due to the diversity of the measured liquids.

### 3.3. Reactance Influence on Resonance of the Quartz Crystal

The series resonant frequency, $f_{s}$, of the quartz crystal without an additionally connected reactance in series is given by:

(5) $$f_{s}=\frac{1}{2\sqrt{L_{1}C_{1}}}\;,$$

where $L_{1}$ and $C_{1}$ are the inductance of the coil and capacitance of the capacitor respectively, in the equivalent quartz crystal scheme (Figure 1b) [36,38,39,40].

The complex impedance, $\underline{Z}$, for the crystal equivalent circuit can be expressed in terms of the normalized frequency $\Omega =\omega /\omega_{0}$, where $\omega_{0}=2\pi f_{s}$ is the resonant angular frequency (for details of derivation see [32,38,41]), as:

(6) $$\underline{Z}=R_{1}\frac{1+j\frac{\omega_{0}L_{1}}{R_{1}}\left(\Omega -\frac{1}{\Omega }\right)}{1+\frac{C_{0}}{C_{1}}\left(1-\Omega^{2}\right)+j\frac{C_{0}}{C_{1}}\frac{R_{1}}{\omega_{0}L_{1}}\Omega }\;.$$

Since the glass test tube with its capacitance $C_{01}$ and resistance $R_{n1}$ is connected in series with the quartz crystal, and the liquid under test can be presented as a capacitor with capacitance $C_{d}$ and a resistor with resistance $R_{d}$ in parallel with $C_{01}$, the complex impedance of the whole circuit (${\underline{Z}}_{T}$) is given by [41]:

(7) $${\underline{Z}}_{T}=R_{1}\frac{1+j\frac{\omega_{0}L_{1}}{R_{1}}\left(\Omega -\frac{1}{\Omega }\right)}{1+\frac{C_{0}}{C_{1}}\left(1-\Omega^{2}\right)+j\frac{C_{0}}{C_{1}}\frac{R_{1}}{\omega_{0}L_{1}}\Omega }+\frac{\frac{\omega_{0}R_{d}}{j\Omega C_{d}}}{\left(\frac{\omega_{0}}{j\Omega C_{d}}+R_{n1}\right)}.$$

The crystal resistance $R_{1}$ and the resistance $R_{n1}$ do not affect the resonant frequency $f_{s}$. By setting $R_{1}$ and $R_{n1}$ in Equation (2) to zero, we find a simplified expression for the impedance (${\underline{Z}}^{*}$) [38,42,43]:

(8) $${\underline{Z}}^{*}=\frac{1}{j\omega C_{d}}\frac{C_{1}+C_{0}+C_{d}-\omega^{2}L_{1}C_{1}\left(C_{0}+C_{d}\right)}{C_{0}+C_{1}-\omega^{2}L_{1}C_{1}C_{0}}\;.$$

By setting ${\underline{Z}}^{*}$ to zero, a new resonant frequency, $f_{s}{}^{*}$, is obtained:

(9) $$f_{s}{}^{*}=\frac{1}{2\pi \sqrt{L_{1}C_{1}}}\sqrt{1+\frac{C_{1}}{C_{0}+C_{d}\;}}\;.$$

**Figure 2:**
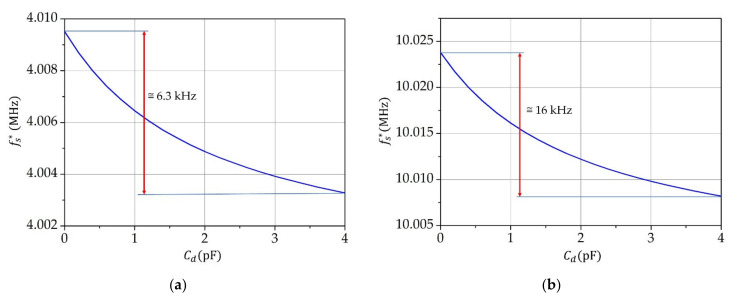
Resonant frequency $f_{s}{}^{*}$ as a function of capacitance $C_{d}$ for: (**a**) 4 MHz and (**b**) 10 MHz crystal.

Equation (9) reflects the change of frequency $f_{s}{}^{*}$ within the range of variation of capacitance $C_{d}$. Figure 2a,b illustrate the changes of the resonant frequency $f_{s}^{*}$ (Equation (9)) for 4  and 10 MHz quartz crystals. A 10 MHz crystal displays an approximately 2.5 times higher frequency sensitivity than the 4 MHz crystal at the same change of capacitance $C_{d}$.

A change of capacitance $C_{d}$ triggers a change of the oscillator’s frequency $f_{osc1}$ in the range between 1 and 20 kHz. Modulation of two frequencies ($f_{osc1\;}$ and $f_{ref}$) leads to the formation of a triangular signal on a low-pass filter, with resistance R and capacitance C, which is then changed by the Shmitt circuit (to reduce interference when the frequency, $f_{out}$, is measured) into a square signal [32,36,37,38,42], with frequency $f_{out}$:

(10) $$f_{out}=\left[\left(f_{osc1}+df_{osc1}\left(T\right))-(f_{ref}+df_{ref}\left(T\right)\right)\right]+df_{c_{err}}\;.$$

The temperature changes in the frequencies of the detection and reference oscillator ($df_{osc1}\left(T\right)$ and $df_{ref}\left(T\right)$) are not fully compensated because the temperature–frequency characteristics of the two oscillators are not exactly equal. The HM 8123 counter frequency measurement error ($df_{c\_err}$) represents the frequency instability of the counter, which equals $\pm 1.0\times 10^{-8}$ in the temperature range 0–50 °C [32,36,38,42].

**Figure 3:**
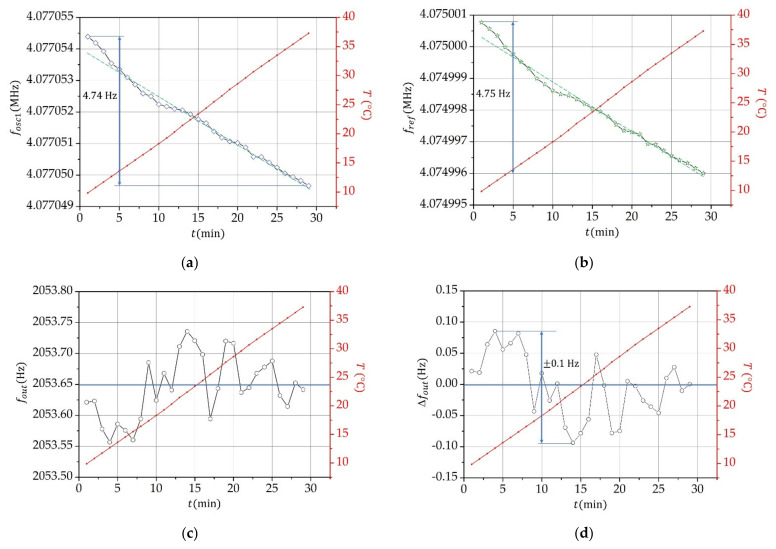
Oscillator’s frequency variation in relation to the temperature variation (10–40 °C). The temperature was increased uniformly from 10–40 °C in a time span of 30 min (red dashed line). (a,b) Variation of the oscillator frequencies $f_{osc1}$ and $f_{ref}$. The green dashed line presents the trend. (c) Frequency difference, $f_{out}$, between the two oscillators. (d) Variation of the frequency difference, $\Delta f_{out}$, between both oscillators in relation to the mean value of 2053.65 Hz (blue line in Figure 3c).

Figure 3a,b illustrate variation of the frequencies $f_{osc1}$ and $f_{ref}$ due to the temperature change of the experimental setup in the range of 10–40 °C (in a climate test chamber, Weiss Technik). We observe that a linear increase in temperature leads to an almost linear decrease in the frequencies of the detection and reference oscillator. Figure 3c shows a temperature variation of the difference of frequencies of the detection and reference oscillator: $f_{out}=f_{osc1}-f_{ref}$. Within the temperature range from 10 to 40 °C, the mean value of the frequency $f_{out}$ is 2053.65 Hz, when the test tube is empty. The dynamic variation of $\Delta f_{out}$ in relation to the mean value of $f_{out}$ is shown in Figure 3d: the frequency varies in the range of ±0.1 Hz.

### 3.4. Permittivity Measurements by Using Capacitive-Dependent Quartz Crystals

The measured complex permittivity depends both on the oscillators’ frequency $f_{osc1}$ change triggered by the change of capacitance $C_{d}$, and on the conductance change due to the resistance $R_{d}$ (Figure 1c). The test tube filled with the measured liquid put between the capacitor plates fills the capacitor partially by glass and partially by the measured liquid. The capacitance of such an arrangement equals to the capacitance of three capacitors in series: one capacitor filled by glass of capacitance $C_{s}$, then a capacitor filled by the measured liquid with capacitance $C_{l}$, and finally, another capacitor filled by glass and capacitance $C_{s}$. The equivalent capacitance of such an arrangement is:

(11) $$C_{d}=\frac{C_{s}C_{l}}{2C_{l}+C_{s}}\;.$$

If the test tube is empty, then $C_{l}=C_{air}$, and $C_{d}$ as given by Equation (11) is the capacitance of an empty test tube, and because $C_{s}\gg C_{air}$, $C_{d0}≅C_{air}$. When the measured liquid is filled in the test tube, $C_{l}=\varepsilon_{r}C_{air}$. Thus, the relative permittivity of the measured liquid can be expressed from Equation (11) as:

(12) $$\varepsilon_{r}=\frac{C_{d}C_{s}}{\left(C_{s}-2C_{d}\right)C_{air}}\;,$$

which in the case of $C_{s}\gg C_{d}$, reduces to:

(13) $$\varepsilon_{r}=\frac{C_{d}}{C_{d0}}\;.$$

The capacitance $C_{d}$ is related to the measured frequency. From Equation (9), we find:

(14) $$C_{d}=\frac{C_{1}}{{\left(\frac{f_{s}{}^{*}}{f_{s}}\right)}^{2}-1}-C_{0}\;.$$

The expression in Equation (13) is valid only for liquids with low relative permittivity. We can estimate the values of $C_{s}$, $C_{l}$ and $C_{d}$ by assuming a parallel plate capacitor with the distance between the plates being equal to the test tube diameter (12 mm), with a layer of glass next to each plate (1 mm thick, relative permittivity 8.3, see Table 2) and the liquid in between (10 mm thick). With the area of the capacitor plates being $2.5\;{\mathrm{cm}}^{2}$, we find $C_{s}\approx 19\;\mathrm{pF}$, $C_{air}\approx 0.23\;\mathrm{pF}$, $C_{d0}\approx C_{air}$, for the test tube filled with benzene ($\varepsilon_{r}\approx 2.3$) $C_{d}\approx 0.5\;\mathrm{pF},$ and if filled with water ($\varepsilon_{r}\approx 80$), $C_{d}\approx 6\;\mathrm{pF}$. Since the capacitor plates are curved, the effective distance between the plates is lower than that taken in the above estimate, so all the capacitances are systematically larger. This is not of crucial importance. In fact, there are also other parasitic capacitances in the circuit that one cannot avoid. The problem is solved by the calibration of the sensor.

The dielectric loss is obtained as a ratio between the conductance of the tube with and without the liquid:

(15) $$\varepsilon^{\prime \prime }=\frac{G_{d}}{\omega C_{d0}}=\frac{G_{d}}{2\pi f_{s}{}^{*}C_{d0}}\;.$$

**Figure 4:**
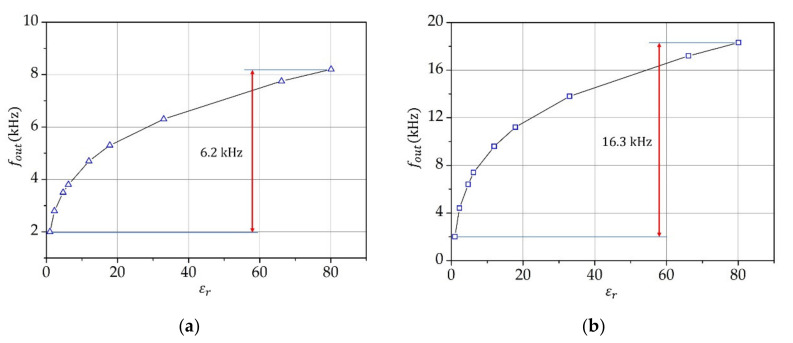
Calibration of measurements of relative permittivity by using pure liquids given in Table 3 at 20 °C at the oscillator frequency (**a**) $f_{osc1}=4\;\mathrm{MHz}$ ($Q\;\mathrm{data}:\;C_{1}=10\;\mathrm{fF}$, $L_{1}=158.314\;\mathrm{mH}$, $C_{0}=2\;\mathrm{pF}$) and (**b**) $f_{osc1}=10\;\mathrm{MHz}$ (Q data: $C_{1}=10\;\mathrm{fF}$, $L_{1}=25.33\;\mathrm{mH}$, $C_{0}=2\;\mathrm{pF}$).

Figure 4 shows the calibration curves for the measuring system at 20 °C. They were obtained by using liquids listed in Table 3, providing that the relative permittivity is measured at low frequencies where no relaxation effects occur. Table 3 lists the values of relative permittivity of the used liquids at 20 °C and standard atmospheric pressure obtained from the CRC Handbook of Chemistry and Physics [44].

### 3.5. Measurements of Conductance of Liquids

The conductance is measured by measuring the voltage on the resistor with a known resistance, $R_{n1}$ (Figure 1). The voltage signal is amplified by the amplifier $O_{p}$, then transformed by a full-wave rectifier (block $R_{e}$) and integrated ($I_{int}$) to obtain the average value of the voltage, ${\bar{U}}_{\sigma }$, which is digitized in a microcontroller (μC—NXP LPC 1768) by a 12-bit A/D converter.

To obtain the conductance of the liquid ($G_{d}$), we measure the voltage on $R_{n1}$ in the case of an empty glass test tube ($U_{e}$) and in the case of the liquid in the test tube ($U_{d}$). The current flowing through $R_{n1}$ is then $I_{e}=U_{e}/R_{n1}$ and $I_{d}=U_{d}/R_{n1}$ in the case of the empty and filled test tube, respectively. The joint impedance ($Z_{d}^{*}$) of the test tube with liquid together with the measuring resistor is:

(16) $$Z_{d}^{*}=R_{n1}+{\left(\frac{1}{R_{d}}+j\omega C_{d}\right)}^{-1}$$

and if the test tube is empty, it is:

(17) $$Z_{e}^{*}=R_{n1}-j\omega C_{d0}\;.$$

Thus, the ratio between the currents $I_{d}$ and $I_{e}$ flowing through the resistor $R_{n1}$ in the case of the filled and empty test tube is:

(18) $$\frac{I_{d}}{I_{e}}=\frac{\sqrt{1+\omega^{2}C_{d}^{2}R_{d}^{2}}}{\omega C_{d0}R_{d}}\;,$$

where we have neglected terms with $R_{n1}$, because this resistance is expected to be much lower than $R_{d}$. From Equation (17), we express the conductance $G_{d}=R_{d}^{-1}$ of the liquid:

(19) $$G_{d}=\omega C_{d0}\sqrt{{\left(\frac{U_{d}}{U_{e}}\right)}^{2}-{\left(\frac{C_{d}}{C_{d0}}\right)}^{2}}\;,$$

where we used $I_{d}/I_{e}=U_{d}/U_{e}$. In the case of $\omega \left(C_{10}+C_{d}\right)R_{d}\ll 1$, Equation (18) simplifies to:

(20) $$G_{d}=\omega C_{d0}\frac{U_{d}}{U_{e}}\;.$$

As glass is not a perfect isolator, parasitic conductance is also present (included in Equation (11) for the capacitance $C_{d0}$) as well as parasitic conductance measured through the voltage $U_{e}$, the effect of which is reduced due to the ratio of voltages in Equation (20).

## 4. Discussion

The usual drawbacks of the capacitance methods used to measure permittivity of liquids result from the fringing-fields effect, electrode polarization, temperature compensation, small sensitivity at capacitance of the order of magnitude 1 pF or lower and the measurement uncertainty, which is of the order of 1%.

By the method described in this article, the resonant frequency of a quartz oscillator is measurably affected by a fF change in the capacitance of the sample. The detection electrodes of the capacitor $C_{01}$ (Figure 1) block the electrode polarization effect as they do not have a galvanic contact with the liquid. In order to reduce the fringing-fields effect as much as possible, the shield electrodes on the glass test tube must be placed in the immediate vicinity of the detection capacitor and grounded (Figure 1a). The comparison of sensitivity (Figure 2) shows the influence of capacitance on the serial resonant frequency and illustrates that the quartz crystal has an approximately 2.5 times higher sensitivity at 10  than at 4 MHz.

The temperature compensation of the suggested capacitive-dependent quartz crystal method is created by using two quartz oscillators that are made in the same way (with elements of the same quality) and have a temperature–frequency pair of quartz crystals. When two oscillators and a temperature–frequency pair of crystals that have as similar characteristics as possible are used, the temperature compensation is in the range below 0.1 ppm (Figure 3). Changes in pressure and humidity do not affect the measurements, because the electronic circuit is built symmetric and the sensor is enclosed in a housing (as opposed to the use of quartz for the quartz crystal tuning fork, where the sensor is exposed to the environment [45]).

At the same time, when the frequency $f_{out}$ is measured with the help of a reference oscillator, the measurements are more accurate, because the frequency difference is in the kHz region, which also enables further processing of the signal by a microcontroller.

The long-term stability of the detection and reference oscillators depends on the drive levels of both. The drive levels should be kept at a minimum level at which the oscillation is initiated and maintained—it should be less than half of the maximum drive level (in our case, the drive level is 50 μW). An excessive drive may cause a frequency drift, spurious emissions, “ringing” wave forms, excessive ageing and/or fatal structural damage to the crystal [46].

The currently available oscillometric measurements are used to study dielectric properties of nonionic mixtures of liquids, where the dielectric behavior predominates (e.g., ethanol/nitrobenzene, benzene/chlorobenzene and alcohol/water). The major advantage of the proposed method is that, in addition to the dielectric component, the conductivity of ionic liquids can also be measured with high sensitivity and accuracy.

The calibration of the measuring system was made by using liquids from Table 3 at 20 °C. The uncertainties of the proposed measurement method are: positioning of capacitors (0.01%), glass tube nonlinearity (0.01%), temperature compensation of oscillators (±0.05 Hz), quartz temperature–frequency characteristics (0.02 ppm), frequency measurement (0.0001 Hz), calibration (0.05%) and linearization of characteristics (0.05%). By taking into account all of the above-mentioned uncertainties and the dynamic measurement due to the change of temperature in the range of 20 ± 2 °C, the uncertainty of the capacitive-dependent quartz crystal method is in the range below ±0.05%, which represents a significant reduction of the uncertainty of the proposed method when compared to the standard capacitive method (see Table 1).

## 5. Conclusions

The research discussed in this article presents an improved way of measuring complex permittivity of liquids by using the capacitive-dependent quartz crystal method and two quartz oscillators. The proposed method demonstrates high sensitivity, temperature compensation and an improved measurement uncertainty in the range of ±0.05%. Based on the performance of the proposed method in the experimentally measured liquid samples, the method may be advantageous when measuring the relative permittivity and conductivity of liquids with high precision, which is of special importance in the field of power engineering to monitor the quality of oils, liquid fuels, lubricants, solvents, etc.

## Figures and Tables

**Table 1:** Advantages of different dielectric measurement methods and their typical uncertainties of the relative permittivity ($\Delta \varepsilon_{r}/\varepsilon_{r}$) and loss tangent (tanδ) [1].

Technique	Advantage	$\Delta \varepsilon_{r}/\varepsilon_{r}\;(\%)$	$\Delta \left(\tan\delta \right)$
Coaxial line, waveguide	Broadband	±1 to 10	±0.005
Slot in waveguide	Broadband	±1 to 10	±0.005
Capacitor	Low frequency	±1	±5 $\times 10^{-4}$
Cavity	Very accurate	±0.2	±5 $\times 10^{-5}$
Dielectric resonator	Very accurate	±0.2	±1 $\times 10^{-5}$
Coaxial Probe	Non-destructive	±0.2 to 10	±0.02
Fabry-Perot	High frequency	±2	±0.0005

**Table 2:** The relative permittivity ($\varepsilon_{r}$) and loss tangent (tanδ) for different materials of a glass test tube at various frequencies (at 20 °C) [33].

Material		100 kHz	10 MHz	100 MHz
E-glass	$\varepsilon_{r}$ tanδ	6.39 0.0027	6.32 0.0015	6.22 0.0023
Fused quartz	$\varepsilon_{r}$ tanδ	3.78 0.00075	3.78 0.0002	3.78 0.0001
Fused silica	$\varepsilon_{r}$ tanδ	3.78 0.00011	3.78 0.00001	3.78 0.00003
Iron-sealing glass	$\varepsilon_{r}$ tanδ	8.38 0.0004	8.30 0.0005	8.20 0.0009

**Table 3:** The relative permittivity ($\varepsilon_{r}$) of liquids used for calibration, measured in static fields or at low frequencies at 20 °C and standard atmospheric pressure [44].

Mol. Form.	Liquid	$\varepsilon_{r}$
C_6_H_6_	Benzene	2.2825
C_4_H_11_N	Butylamine	4.71
C_2_H_4_O_2_	Acetic acid	6.20
C_7_H_14_O	2-Heptanone	11.95
C_4_H_10_O	1-Butanol	17.84
CH_4_O	Methanol	33.00
C_4_H_6_O_3_	Propylene carbonate	66.14
H_2_O	Water	80.100
